# Book Notices

**Published:** 1864

**Authors:** 


### Book Notices.

The following valuable new works have been received, and shall be noticed more fully as soon as our time will permit an adequate examination:—

The Principles and Practice of Obstetrics. Illustrated with 159 lithographic figures from original photographs, and with numerous woodcuts. By Hugh L. Hodge, M.D., Emeritus Prof. of Obstetrics, and Diseases of Women and Children, in the University of Pennsylvania; &c., &c., &c. Philadelphia: Blanchard & Lea. 1864.

Medical Diagnosis with Special References to Practical Medicine. A Guide to the Knowledge and Discrimination of Diseases. By J. M. DaCosta, M.D., Lecturer on Clinical Medicine and Physician to the Philadelphia Hospital; &c., &c. Philadelphia: J. B. Lippincott & Co. 1864.

A Manual of the Practice of Medicine. By Thomas Hawkes Tanner, M.D., F.L.S., &c., &c., &c. From the last London edition, enlarged and improved. Philadelphia: Lindsay & Blakiston, 1864.

The Medical Register of the City of New York, for the year 1864. By Guido Furman, M.D. Published under the auspices of the "New York Medico-Historical Society." New York: Edward O. Jennings, Printer.

The above works were received through the well-known Bookstore of W. B. Keen & Co., 148 Lake Street, Chicago, where they are for sale.

The following additional works were received through the

Bookstore of S. C. Griggs & Co., 41 Lake Street, Chicago, where they are for sale:—

Military, Medical, and Surgical Essays, prepared for the United States Sanitary Commission. Edited by William A. Hammond, M.D., Surgeon-General U.S.A. Philadelphia: J. B. Lippincott & Co.

---

The Philosophy of Marriage, in its Social, Moral, and Physical Relations with the Physiology of Generation in the Vegetable and Animal Kingdoms. By Michael Ryan, M.D., Member of the Royal College of Physicians and Surgeons in London. From the last London edition. Philadelphia: Lindsay & Blakiston. 1864.

---

Alcohol and Tobacco. Alcohol: Its Place and Power. By James Miller. The Use and Abuse of Tobacco. By John Lizars. Philadelphia: Lindsay & Blakiston. 1864.

---

Theory and Practice of the Movement Cure: or the Treatment of Curvatures, Deformities, &c., &c., by the Sweedish System of Localized Movements. By Charles Fayette Taylor, M.D.. Second edition; with Illustrations. Philadelphia: Lindsay & Blakiston. 1864.

